# Case Report: Early Association of Vemurafenib to Standard Chemotherapy in Multisystem Langerhans Cell Histiocytosis in a Newborn: Taking a Chance for a Better Outcome?

**DOI:** 10.3389/fonc.2021.794498

**Published:** 2021-12-13

**Authors:** Stefania Gaspari, Valentina Di Ruscio, Francesca Stocchi, Roberto Carta, Marco Becilli, Maria Antonietta De Ioris

**Affiliations:** Department of Hematology/Oncology, Cell and Gene Therapy, Scientific Institute for Research, Hospitalization and Healthcare (IRCCS) Bambino Gesù Children’s Hospital, Rome, Italy

**Keywords:** target therapy, Langerhans cell histiocytosis, Vemurafenib, multisystem disease, newborn

## Abstract

Langerhans cell histiocytosis (LCH) is due to aberrant monoclonal proliferation and accumulation of dendritic cells, ranging from a self-limiting local condition to a rapidly progressive multisystem disease with poor prognosis. Pathogenic cells originate from a myeloid-derived precursor characterized by an activation of the MAPK/ERK signaling pathway in about 70% of cases. In particular, BRAF V600E mutation is usually associated with a more severe clinical course and poor response to chemotherapy. We report on a newborn with multisystem LCH in life-threatening medical conditions. At diagnosis, the patient was successfully treated with the early association of BRAF inhibitor Vemurafenib to standard chemotherapy representing a new approach in first-line treatment. A rapid clinical improvement with a prompt fever regression from day 2 and complete resolution of skin lesions by week 2 were observed; laboratory data normalized as well. Vemurafenib was discontinued after 12 months of treatment. No signs of relapse occurred after 12 months of discontinuation. This case indicates that early combination of target therapy with standard treatment may induce rapid response and prolonged disease remission without significant toxicities in infants. This approach represents a valid and safe option as first-line treatment in multisystem disease, especially in high-risk patients.

## Introduction

Langerhans cell histiocytosis (LCH) is a rare inflammatory myeloid neoplasm, with an incidence ranging from 2.6 to 8.9 cases per million in children under the age of 15 years with a mean age at diagnosis of approximately 3 years (1, 2). LCH is characterized by clonal infiltrating pathological proliferation of CD1a+/CD207+ dendritic cells, spanning from a self-limiting local condition to a rapidly progressive multisystem disease with poor prognosis (3).

Treatment varies greatly depending on the involved organs (4). A watch-and-wait strategy may be appropriate for single-organ disease. A surgical approach is the current option for isolated bone involvement. Skin lesions may resolve spontaneously or may need topical steroids, oral methotrexate, or thalidomide (5). These patients with localized disease usually have an excellent event-free survival (EFS) and overall survival (OS) of 80.6% and 100%, respectively (6, 7).

Multisystem disease (MS) is classified according to the presence of risk organs (RO) involvement, including the hematopoietic system, spleen, and/or liver (4).

Treatment with prednisone and vinblastine, according to LCH IV Protocol (8), represents the standard of care of MS-LCH with a 10-year OS and EFS rates of 49% and 79%, respectively (7, 9, 10). Several studies demonstrated that OS is significantly lower among patients younger than 2 years of age, who frequently show a RO involvement, elevated inflammatory markers, and resistance to standard treatment regimens (11–13). Refractory LCH is usually treated with highly toxic second-line chemotherapy (including cytarabine and vincristine or cladribine) or hematopoietic stem cell transplantation (HSCT).

According to recent molecular findings, a targeted therapy is proposed in high-risk pediatric patients, especially with RO pattern, who fail standard therapy or experience multiple relapses.

Recurrent BRAFV600E mutations have been identified in approximately 60% of LCH patients (14), changing the natural history of the disease. In fact, it is widely known that BRAF gene controls the production of BRAF protein, which is part of a signaling pathway known as the RAS/MAPK pathway, which regulates cell proliferation, differentiation, migration, and apoptosis (14, 15). In the presence of BRAF mutations, the BRAF-mutated protein provides continuous signal to the nucleus and allows uncontrolled growth and differentiation of Langerhans cells (16). Other gene mutations, such as that involving the MAP2K gene and other rarer genes (such as ARAF), have also been described (17).

The identification of BRAF mutation has led to the promising therapeutic approach based on the BRAF inhibitor Vemurafenib (18–20) in particular for infants with MS disease. However, it is known that it failed to eradicate neoplastic clone and to maintain long-term disease remission (21).

In this report, we describe a newborn with MS-LCH diagnosed in life-threatening medical conditions successfully treated since diagnosis with Vemurafenib in combination with standard treatment, with no sign of disease relapse several months after BRAF inhibitor discontinuation.

## Case Description

A 20-day male was referred to primary neonatal intensive care unit in critical conditions with persistent fever, enlarged abdomen, generalized crusted skin purpuric lesions, increased inflammatory markers associated with diarrhea since first days of life, and anasarca. He was born at 37 weeks after a normal pregnancy and, soon after birth, he presented diffuse multiple purpuric lesions. The child needed non-invasive respiratory support with Continuous Positive Airway Pressure (CPAP) and parenteral nutrition. The chest computed tomography (CT) showed multiple bilateral nodules associated with air-space consolidation. Two sets of blood culture specimens were drawn, which yielded *Candida albicans*.

**Figure 1:**
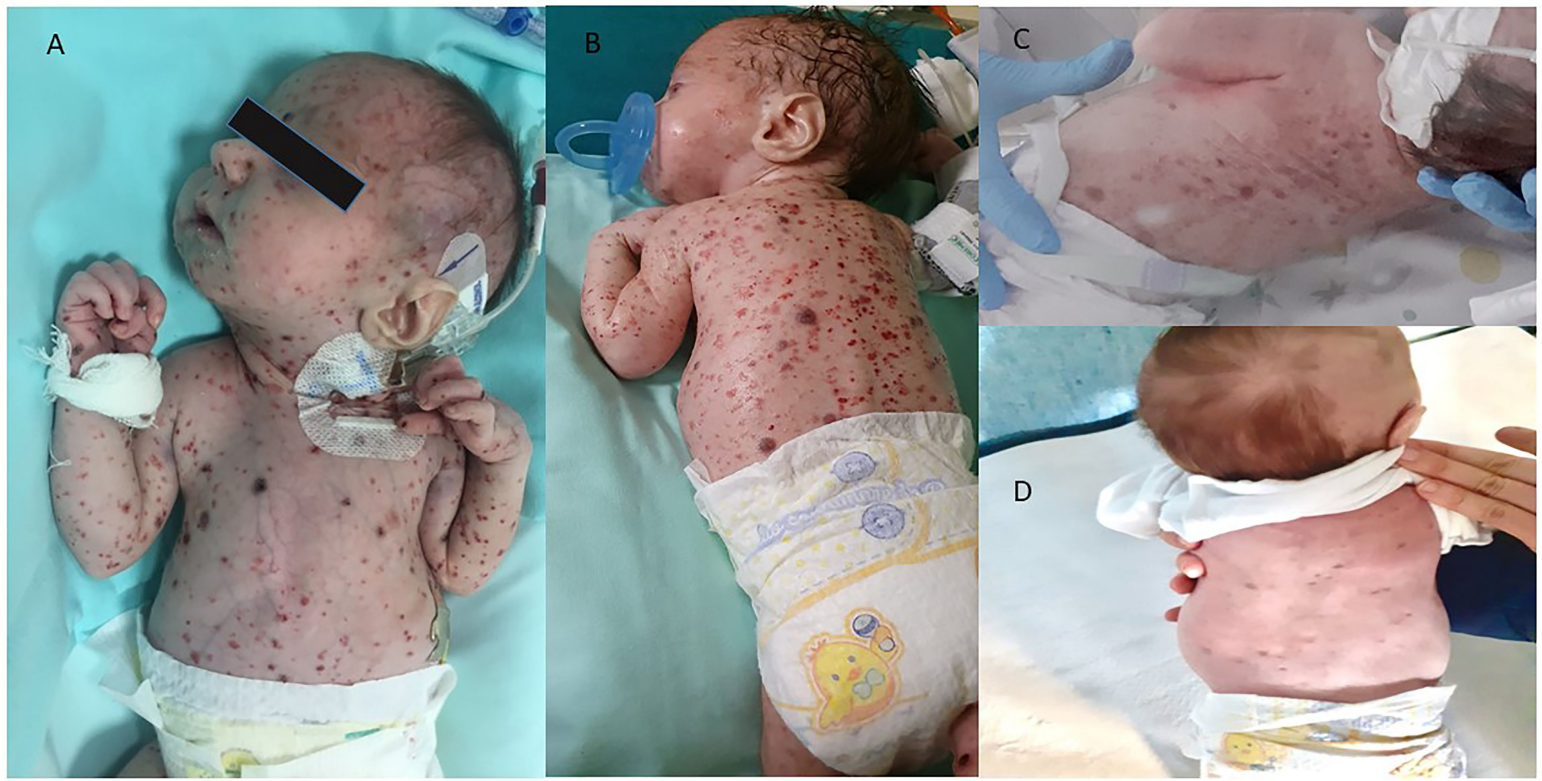
Skin response **(A, B)** skin at diagnosis, **(C)** skin after 2 week of vemurafenib treatment, **(D)** skin after 1 month of vemurafenib treatment.

Clinical examination revealed a purpuric disseminated rash (**Figure 1**), cervical lymphadenopathy, and hepatosplenomegaly. Laboratory findings demonstrated anemia (hemoglobin 7.9 g/dl), thrombocytopenia (26.000/mm^3^), elevated C-reactive protein (11.70 mg/dl), hyperbilirubinemia (total bilirubin 3.07 mg/dl, direct 1.56 mg/dl), with low total protein (3.5 g/dl) and albumin (1.9 g/dl). The child required platelet support almost daily and blood transfusion regularly.

Imaging findings, namely, abdominal ultrasonography and CT scan, revealed hepatomegaly and splenomegaly without focal lesions, as well as multiple bilateral lymphadenopathies in the abdomen and in the thorax.

Magnetic resonance imaging (MRI) of the brain excluded active lesions involving the hypothalamic–pituitary axis, space-occupying lesions, and neurodegenerative lesions of the cerebellum, pons, brainstem, basal ganglia, and gray or white matter of the brain.

Based on the clinical history and laboratory data, a diagnosis of LCH was suspected and confirmed by skin biopsy. The pathology examination revealed multiple aggregates of cells with eosinophilic cytoplasm and nucleus with irregular bland chromatin. CD1a, Langherin, S100, BRAF, and ALKp80 were positive at immunohistochemistry.

A bone marrow biopsy was also obtained, which demonstrated infiltrations of CD1a and CD207-positive histiocytes.

LCH with a multisystem involvement—liver, spleen, bone marrow, lymph nodes, bowel, and skin—was diagnosed with a BRAFV600E mutation detected by immunohistochemical and molecular analysis.

**Figure 2:**
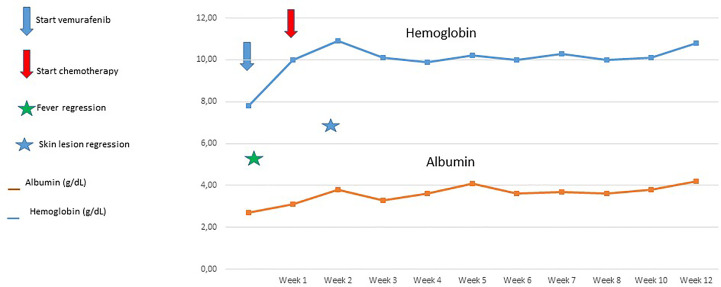
Clinical and laboratory response to the combination of Vemurafenib with chemotherapy.

Considering the life-threatening conditions and the concomitant sepsis due to an opportunistic agent (Candida sepsis), after obtaining parent’s informed consent, the patient started Vemurafenib at a dose of 10 mg/kg twice a day, and after 1 week, the standard treatment with prednisone and vinblastine according to LCH IV Protocol (8) was applied (see **Figure 2**).

After taking Vemurafenib, the patient experienced rapid clinical improvement with prompt fever regression from day 2 and complete resolution of skin lesions by the second week. Within several days, splenomegaly and lymphadenopathies disappeared, liver function normalized, and no further albumin infusions were required. The patient resumed breastfeeding and gradually gained weight; parenteral nutrition support was discontinued on day 10. Blood counts normalized without need of blood-derived products support starting from day 8 (see **Figure 2**).

According to protocol guidelines, chemotherapy with vinblastine and prednisone was administered weekly for the first 12 weeks and then every 3 weeks, without major toxicity. No infectious complications or organ toxicities were recorded. After 12 months of treatment, Vemurafenib was discontinued safely, without disease recurrence. At the last follow up, 24 months after diagnosis, the patient is in good clinical condition and complete remission, with weight and length at the 75th percentile for age.

## Discussion

This is the first report on early administration of the BRAF inhibitor Vemurafenib as a front-line treatment in combination with standard chemotherapy in a newborn with MS-LCH. To the best of our knowledge, Vemurafenib has been employed only in second- or third-line treatment (21–23).

A newborn with severe MS disease and in poor clinical condition represents a critical emergency situation that did not allow us to start chemotherapy treatment safely, also considering the recent history of Candida sepsis. The target therapy with Vemurafenib resulted in a brilliant clinical response with fever regression, rapid improvement of skin lesions, and normalization of biological markers of systemic inflammation.

The discovery of recurrent BRAF mutations is a real breakthrough in understanding LCH pathogenesis (12, 14), with the possibility of selective inhibition of the BRAF kinase with Vemurafenib (22, 24, 25).

In relapsed/refractory LCH, Vemurafenib alone is able to induce either complete or partial response in 70% and 30% of children, respectively, with less toxicities than cladribine, cytarabine, or allogeneic HSCT, a procedure that represents the current standard treatment for refractory LCH. Despite the high rate of response associated with the use of Vemurafenib, a high proportion of disease relapse is reported after drug discontinuation (23).

Heritier et al. first reported an 8-month-old infant with multisystem-resistant LCH receiving Vemurafenib for a 3-month period as a second-line treatment. Thereby, 2 months after discontinuation, a skin relapse was detected and Vemurafenib was resumed (24).

In our case, Vemurafenib was administered at diagnosis as a front-line, single-agent treatment, resulting in prompt reduction of disease burden and clinical and biological response.

The combination of Vemurafenib with LCH standard chemotherapy after the first week, never reported before, was well tolerated, and caused no major side effects. In order to prevent disease reactivation, BRAF inhibitor and vinblastine were maintained for a 12-month period. After a follow-up of 12 months from Vemurafenib discontinuation, the patient is in good clinical condition (Lansky scale 100% and at 75th centile for length and weight) and in complete remission with no signs of disease recurrence.

In conclusion, our case demonstrates that the early combination of target therapy with standard chemotherapy is effective in inducing a prompt response and prolonged disease remission with no toxicities. Therefore, we suggest that target therapy associated with standard treatment may be considered as a valid and safe option in first-line treatment in multisystem disease, especially in high-risk patients. Moreover, it should represent a promising option in infants reducing the chemotherapy use. Prospective trials are warranted to evaluate the effectiveness and the safety of BRAF inhibitor in combination with standard treatment as front-line therapy.

## Data Availability Statement

The original contributions presented in the study are included in the article/supplementary material. Further inquiries can be directed to the corresponding author.

## Author Contributions

All the authors took care of the patient. All authors contributed to the article and approved the submitted version.

## Conflict of Interest

The authors declare that the research was conducted in the absence of any commercial or financial relationships that could be construed as a potential conflict of interest.

## Publisher’s Note

All claims expressed in this article are solely those of the authors and do not necessarily represent those of their affiliated organizations, or those of the publisher, the editors and the reviewers. Any product that may be evaluated in this article, or claim that may be made by its manufacturer, is not guaranteed or endorsed by the publisher.
